# LRRK2 activity does not dramatically alter α-synuclein pathology in primary neurons

**DOI:** 10.1186/s40478-018-0550-0

**Published:** 2018-05-31

**Authors:** Michael X. Henderson, Chao Peng, John Q. Trojanowski, Virginia M. Y. Lee

**Affiliations:** Department of Pathology and Laboratory Medicine, Institute on Aging and Center for Neurodegenerative Disease Research, University of Pennsylvania School of Medicine, 3600 Spruce St, 3rd Floor Maloney, Philadelphia, PA 19104-4283 USA

**Keywords:** LRRK2, Synuclein, pS129, Aggregates, Inhibitor, G2019S

## Abstract

Mutations in leucine-rich repeat kinase (LRRK2) are the most common cause of heritable Parkinson’s disease (PD), and the most common mutations in LRRK2 lead to elevated kinase activity. For these reasons, inhibitors targeting LRRK2 have been the subject of intense research and development. However, it has been difficult to develop preclinical models that recapitulate PD-relevant LRRK2 phenotypes. The primary pathology in PD is the Lewy body (LB), which is a cytoplasmic aggregate of α-synuclein. The recent demonstration that LB-like aggregates of α-synuclein can be induced in primary neurons has provided a robust model for testing genetic modifiers of PD-relevant aggregation and neurodegeneration. In this study, we test the modulation of α-synuclein pathology by LRRK2 in primary neuron cultures using biochemistry and immunocytochemistry. We find that expression of familial mutant G2019S LRRK2 does not dramatically elevate the pathological burden of α-synuclein or neurodegeneration in neurons. We further test three LRRK2 inhibitors in two strains of wildtype neurons and find that even robust LRRK2 inhibition is insufficient to reduce α-synuclein pathology. LRRK2 inhibitors similarly had no effect in neurons with α-synuclein pathology seeded by human brain-derived pathological α-synuclein. Finally, we find that this lack of pathological modulation by LRRK2 was not confined to hippocampal neurons, but was also absent in midbrain dopaminergic neuron cultures. These data demonstrate that LRRK2 activity does not have more than minor effects on α-synuclein pathology in primary neurons, and more complex models may be needed to evaluate the ability of LRRK2 inhibitors to treat PD.

## Introduction

Parkinson’s disease (PD) is the most common neurodegenerative movement disorder. Patients with this disease experience rigidity, resting tremors, and slowness of movement; 80% of patients will develop dementia throughout the disease course [15]. The clinical diagnosis of PD is confirmed post-mortem by the presence of intracytoplasmic inclusions termed Lewy bodies (LBs), which consist primarily of the synaptic protein α-synuclein [1, 29, 30]. While α-synuclein is thought to be pathogenic in this largely sporadic disease, mutations in several genes can increase the lifetime risk of developing disease. The most commonly mutated gene in hereditary PD is leucine-rich repeat kinase 2 (LRRK2, [11]).

LRRK2 is a widely expressed protein with unclear functions. It has kinase, GTPase and scaffolding domains and has been implicated in intracellular trafficking and cytoskeletal modeling [17]. LRRK2 mutations are found in 4% of hereditary and 1% of sporadic PD patients, and patients with LRRK2 mutations phenocopy sporadic PD patients, with similar onset of disease, α-synuclein pathology, and responsiveness to dopamine replacement [11]. The most common mutation (G2019S) in the kinase domain as well as mutations in the GTPase domain of LRRK2, elevate kinase activity of LRRK2 [8, 26, 31, 36], making LRRK2 an ideal candidate for small molecule inhibitor development. A large number of LRRK2 inhibitors have been developed and refined for increased potency and low off-target effects over the past decade. However, the lack of a reliable preclinical disease model for LRRK2 dysfunction has been a major challenge for validating the therapeutic potential of LRRK2 inhibitors.

To understand the relationship between LRRK2 and α-synuclein and develop a model of LRRK2-dependent PD phenotypes, early studies crossed mice expressing wildtype or G2019S LRRK2 mice with mice overexpressing α-synuclein with the familial A53T mutation. These studies all found little or no effect of LRRK2 expression on α-synuclein pathology [4, 14, 20]. However, later studies which injected adeno-associated virus into the substantia nigra of LRRK2 knockout or G2019S LRRK2 rats found that LRRK2 depletion rescued α-synuclein-induced neurodegeneration [5] and G2019S LRRK2 exacerbated degeneration [3]. Several cell culture studies have found that LRRK2 may directly interact with α-synuclein [9] or indirectly influence α-synuclein expression [23, 28], however none of these cell culture models recapitulate the phosphorylated, detergent-insoluble α-synuclein seen in PD patients. The recent finding that α-synuclein pathology can be induced in wildtype primary neurons by introduction of small amounts of recombinant α-synuclein pre-formed fibrils (PFFs) has allowed the study of α-synuclein pathology in neurons expressing wildtype or mutated LRRK2 [32]. A primary neuron model in which LRRK2 activity modulates α-synuclein pathology would be ideal for screening LRRK2 inhibitors and providing biological rationale for moving toward clinical intervention. We used robust immunocytochemical and biochemical assays for α-synuclein pathology in primary hippocampal and midbrain dopaminergic neurons derived from two strains of wildtype mice and mice expressing the LRRK2 with the familial G2019S mutation to test LRRK2 activity-dependent phenotypes. We induce α-synuclein pathology with addition of both recombinant and human-derived fibrillar α-synuclein. In order to manipulate LRRK2 activity, we used three validated LRRK2 inhibitors. In no system and by no measure did we observe more than very mild LRRK2-dependent changes in α-synuclein pathology.

## Materials and methods

### Animals

All housing, breeding, and procedures were performed according to the NIH Guide for the Care and Use of Experimental Animals and approved by the University of Pennsylvania Institutional Animal Care and Use Committee. C57BL/6 J (NTG, JAX 000664, RRID: IMSR_JAX:000664) and B6.Cg-Tg(Lrrk2*G2019S)2Yue/J (G2019S, JAX 012467, RRID: IMSR_JAX:012467) mice were obtained from Andrew West (University of Alabama, Birmingham). The G2019S mice have multiple inserts of the gene, but were heterozygous at these loci. Subsequently, mice were bred to homozygosity at loci as determined by quantitative PCR and outbreeding. The expression level of G2019S LRRK2 was thereby stabilized in this line of mice. All experiments shown use homozygous G2019S mice. CD1 (Strain 022, RRID: IMSR_CRL:22) mice were obtained from Charles River, Wilmington, MA.

### α-Synuclein PFFs

Purification of recombinant α-synuclein and generation of α-synuclein PFFs was conducted as described elsewhere [21, 33, 34]. For treatment of neurons, α-synuclein PFFs, which were generated at a concentration of 5 mg/mL were vortexed and diluted with Dulbecco’s phosphate-buffered saline (DPBS) to 100 μg/mL. They were then sonicated on high for 10 cycles of 30 s on, 30 s off (Diagenode Biorupter UCD-300 bath sonicator). α-Synuclein PFFs were then diluted in neuron media to 5 μg/mL and added to neuron cultures at a concentration of 1 μg/mL for immunocytochemistry experiments and 2.5 μg/mL for biochemistry experiments.

### Primary hippocampal cultures

Primary hippocampal neuron cultures were prepared from postnatal day (P) 1 NTG and G2019S mice and embryonic day (E) 16–18 CD1 embryos. Dissociated hippocampal neurons were plated at 17,000 cells/well (96-well plate) or 1,000,000 cells/well (6-well plate) in neuron media (Neurobasal medium (ThermoFisher 21,103,049) (or Neurobasal A medium (Thermo Fisher 10,888–022) for postnatal cultures) supplemented with B27 (ThermoFisher 17,504,044), 2 mM GlutaMax (ThermoFisher 35,050,061), and 100 U/mL penicillin/streptomycin (ThermoFisher 15,140,122). LRRK2 inhibitors PF-475 and PF-360 were synthesized at Pfizer, Inc. MLi-2 was obtained from Tocris Bioscience (5756). All LRRK2 inhibitors were reconstituted at 10 mM in DMSO and stored at − 20 °C. There were further diluted to the final concentration indicated in neuron media with DMSO as a vehicle control. Neurons were either fixed with 4% paraformaldehyde, 4% sucrose in phosphate-buffered saline for immunocytochemistry or scraped for biochemistry.

### Primary midbrain/striatum co-cultures

The ventral mesencephalon and striatum were dissected from P2 NTG and G2019S mice in Hibernate A medium (ThermoFisher A1247501) with B27 (ThermoFisher 17,504,044) and 0.5 mM GlutaMax (ThermoFisher 35,050,061). The tissue was then digested in papain (Worthington Biochemical LS003126) for ~ 20 min at 37 °C. Cells were mixed at a ratio of 1:1 and plated at a density of 34,000 cells/well in 96-well black-walled plates (Perkin Elmer 50–905-1605) in Neurobasal A medium (Thermo Fisher 10,888–022) supplemented with B27 and 0.4 mM GlutaMax, 50 ng/mL BDNF (PeproTech 450–02) and 30 ng/mL GDNF (Millipore Sigma GF030).

### Neuron sequential detergent fractionation

Proteins from primary neuronal cultures treated with PBS or α-synuclein PFFs were sequentially extracted as described previously [21, 33, 34]. Briefly, neurons were scraped into 1 volume 1% TX-100 buffer, sonicated and spun at 100,000 x *g* for 30 min. The pellet was sonicated and again spun at 100,000 x *g* for 30 min in 1 volume 1% TX-100 solution to remove remaining TX-100-soluble proteins. This pellet was suspended in 0.5 volumes 2% SDS solution, sonicated and spun once more at 100,000 x *g* for 30 min. The first and final supernatant were kept for immunoblot analysis. Western Blot analysis was performed with primary antibodies targeting α-synuclein (SNL-4, CNDR, 1:10,000), pS129 α-synuclein (ab168381, Abcam, 1:1000), LRRK2 (3514–1, Epitomics, RRID: AB_10643781, 1:2000 or ab133474, Abcam, RRID: AB_2713963, 1:500), pS935 LRRK2 (ab133450, Abcam, 1:500), p62 (H00008878-M01, Abnova, RRID: AB_437085, 1:1000) or GAPDH (2-RGM2, Advanced Immunological, 1:5000). Primary antibodies were detected using IRDye 800 (Li-cor 925–32,210) or IRDye 680 (Li-cor 925–68,071) secondary antibodies, scanned on Li-cor Odyssey Imaging System and analyzed using Image Studio software. Values obtained from this program were normalized to average PFF alone values.

### Human brain sequential detergent fractionation

Frozen postmortem human cingulate gyrus or frontal cortex brain tissue containing abundant α-synuclein-positive inclusions was selected for sequential extraction based on IHC examination of these samples as described [16] using previously established methods. These brains were sequentially extracted with increasing detergent strength as previously published [10]. After thawing, meninges were removed and gray matter was carefully separated from white matter. Gray matter was weighed and suspended in four volumes (*w*/*v*) high salt (HS) buffer (50 mM Tris-HCL (pH 7.4), 750 mM NaCl, 10 mM NaF, 5 mM EDTA, protease and phosphatase inhibitors), followed by homogenization with a dounce homogenizer and centrifugation at 100,000 x *g* for 30 min. The HS wash was repeated and the resulting pellet was then homogenized with 9 volumes HS buffer with 1% TX-100 and centrifuged at 100,000 x *g* for 30 min. The pellet fraction was then homogenized with 9 volumes HS buffer with 1% TX-100 and 30% sucrose and centrifuged at 100,000 x *g* for 30 min to float away the myelin, which was discarded. The pellet was then homogenized with 9 volumes HS buffer with 1% Sarkosyl, rotated for 1 h at room temperature and centrifuged at 100,000 x *g* for 30 min. The resulting pellets were washed once with Dulbecco’s PBS and re-suspended in Dulbecco’s PBS by brief sonication (QSonica Microson™ XL-2000; 20 pulses; setting 2; 0.5 s/pulse). This suspension was termed the “sarkosyl insoluble fraction” containing pathological α-synuclein and used for the cellular assays described here. The amounts of α-synuclein in the sarkosyl insoluble fractions were determined by sandwich ELISA as described previously [2] using Syn9027 (100 ng/well) as the capture antibody and MJF-R1 (1:1000 dilution) as the reporter antibody.

### Immunocyctochemistry

Immunostaining of neuronal cultures was carried out as described previously [12]. Briefly, cells were permeabilized in 3% BSA + 0.3% TX-100 in PBS for 15 min at room temperature. After a PBS wash, cells were blocked for 50 min with 3% BSA in PBS prior to incubation with primary antibodies for 2 h at room temperature. Primary antibodies used were targeting pS129 α-synuclein (81A, CNDR, 1:5000), MAP2 (17028, CNDR, 1:5000), NeuN (MAB377, Millipore, RRID: AB_2298772, 1:1500) or tyrosine hydroxylase (TH, T2928, Sigma-Aldrich, RRID: AB_477569, 1:1000). Cells were washed 5× with PBS and incubated with secondary antibodies for 1 h at room temperature. After 5× wash with PBS, cells were incubated in 1:10,000 DAPI in PBS. 96-well plates were imaged on In Cell Analyzer 2200 (GE Healthcare) and analyzed in the accompanying software. A standard intensity-based threshold was applied to MAP2 and pS129 α-synuclein channels and positive area was quantified. For NeuN quantification, an object-based analysis was applied to identify objects of specified size and intensity. TH+ cell analysis was based on intensity and size of objects (TH+ cells bodies are much more intense than associated processes). All quantification was optimized and applied equally across all conditions.

### Statistical analysis

**Fig. 1:**
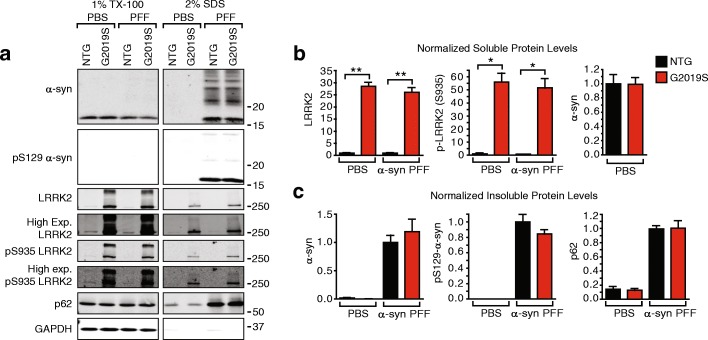
G2019S LRRK2 hippocampal neurons do not have elevated α-synuclein pathology 14 days post-transduction. **a** Primary hippocampal neurons from NTG or G2019S pups were transduced with 2.5 μg/mL α-synuclein PFFs and allowed to age a further 14 days prior to sequential detergent fractionation. TX-100-insoluble α-synuclein and p62 are similar in both neuron types. **b** Quantification of soluble proteins shows ~ 25-fold elevation in the expression of LRRK2 and a commensurate ~ 50-fold elevation in pS395 LRRK2, indicative of the elevated LRRK2 kinase activity associated with the G2019S mutation. Soluble α-synuclein levels were equivalent between the cultures. **c** No significant differences were found between the genotypes in insoluble proteins by an unpaired t-test with Welch’s correction. (*N* = 3 biological replicates for each protein). Means + s.e.m.; **P < 0.01; *P < 0.05 by an unpaired t-test with Welch’s correction for unequal variances. All values are normalized to NTG neurons treated with α-synuclein PFFs and DMSO

**Fig. 2:**
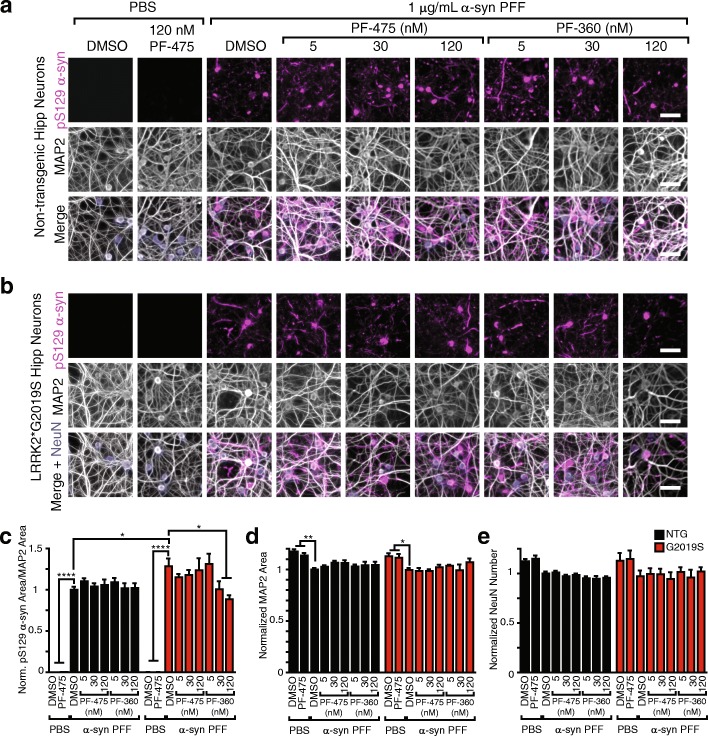
G2019S LRRK2 hippocampal neurons show mild, reversible elevation in induced α-synuclein pathology 21 days post-transduction. Primary hippocampal neurons from NTG (**a**) or G2019S (**b**) pups were transduced with α-synuclein PFFs and allowed to age a further 21 days prior to fixation and staining for pS129 α-synuclein (magenta), MAP2 (gray) and NeuN (blue). The neurons were additionally treated with LRRK2 inhibitors PF-475 and PF-360 2 days prior to transduction and fed with media containing inhibitors each week thereafter. No large differences can be observed in the type or abundance of α-synuclein pathology. **c** Quantification of α-synuclein pathology reveals a mild elevation in G2019S neurons, which is reversible with 30 or 120 nM PF-360. *P < 0.05 by Dunnett’s multiple comparison test between NTG and G2019S neurons or Sidak’s multiple comparisons test between G2019S neurons treated with LRRK2 inhibitors. **d** MAP2 area is reduced with 21 days α-synuclein PFF treatment in both NTG and G2019S neurons, and is not significantly affected by LRRK2 inhibitor treatment. *P < 0.05 by 2-way ANOVA with Dunnett’s multiple comparison test for comparison within genotype. **e** The number of neurons, as quantified by NeuN number, is reduced with 21 days α-synuclein PFF treatment in both NTG and G2019S neurons, although not significantly by 2-way ANOVA followed by Dunnett’s multiple comparison test and is not significantly affected by LRRK2 inhibitor treatment. (*N* = 12 biological replicates). Means + s.e.m.; all values are normalized to NTG neurons treated with α-synuclein PFFs and DMSO. Scale bars = 50 μm

**Fig. 3:**
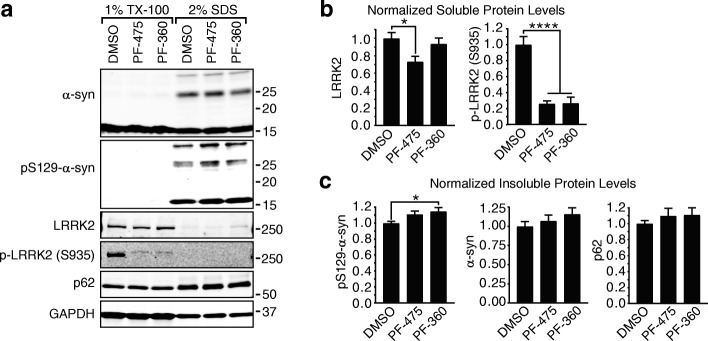
LRRK2 inhibition does not reduce insoluble α-synuclein in wildtype hippocampal neurons. **a** Primary hippocampal neurons from CD1 pups were treated with 30 nM LRRK2 inhibitors PF-475, PF-360 or DMSO as a vehicle control, then transduced with 2.5 μg/mL α-synuclein PFFs and allowed to age a further 14 days prior to sequential detergent fractionation. TX-100-insoluble α-synuclein and p62 are similar in both LRRK2 inhibitor treated and untreated neurons. **b** Quantification of soluble proteins show some reduction of LRRK2 protein levels PF-475 treatment and ~ 75% inhibition of LRRK2 activity (as assayed by pS935 LRRK2) by both PF-475 and PF-360. **c**  Insoluble pS129 α-synuclein was slightly, but significantly elevated by PF-360 treatment, while α-synuclein and p62 were unchanged by one-way ANOVA. (*N* = 5 biological replicates for each protein). Means + s.e.m.; *P < 0.05, ****p < 0.0001 by one-way ANOVA with Dunnett’s multiple comparison test. All values are normalized first to GAPDH, as a loading control, then to DMSO-treated neurons

**Fig. 4:**
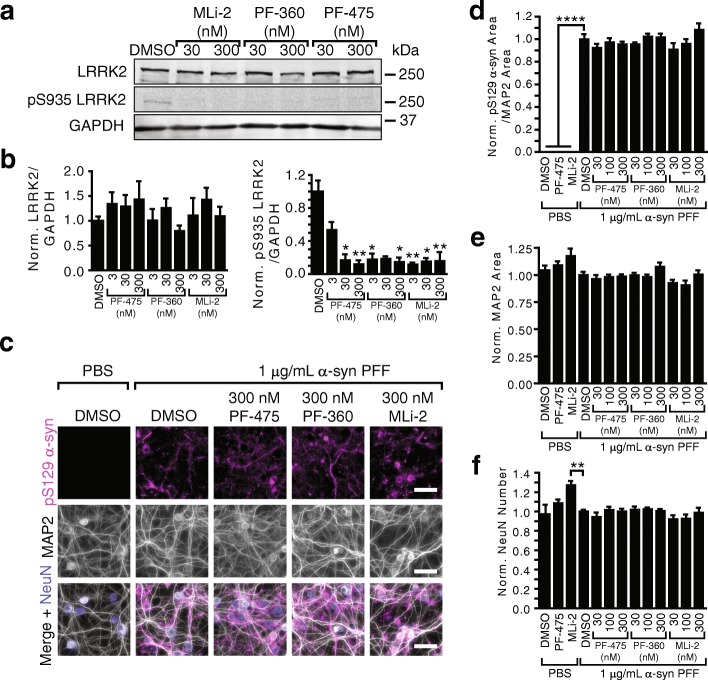
LRRK2 inhibition does not reduce pathological α-synuclein in wildtype hippocampal neurons. **a** Primary cortical neurons were treated with LRRK2 inhibitors PF-475, PF-360, or MLi-2, at 5 DIV and fed with media containing inhibitors each week for 16 days. Cell lysate was run by Western blot to detect LRRK2 and pS935 LRRK2, which is indicative of LRRK2 activity. Images shown are representative of *n* = 4–12 biological replicates. **b** Quantification of Western blot of LRRK2 and pS935 LRRK2. LRRK2 levels were not significantly altered by one-way ANOVA, but pS935 levels were reduced to near undetectable levels (*p < 0.05, **p < 0.01, Kruskal-Wallis test with Dunn’s multiple comparison test). **c** Primary hippocampal neurons from CD1 pups were transduced with α-synuclein PFFs and allowed to age a further 14 days prior to fixation and staining for pS129 α-synuclein (magenta), MAP2 (gray) and NeuN (blue). The neurons were additionally treated with LRRK2 inhibitors PF-475, PF-360, or MLi-2, 2 days prior to transduction and fed with media containing inhibitors each week thereafter. No large differences can be observed in the type or abundance of α-synuclein pathology. **d** Quantification of α-synuclein pathology reveals no change in response to LRRK2 inhibition, while PBS-treated neurons have no pathology (****p < 0.0001 by Dunnett’s multiple comparison test). MAP2 area (**e**) and neuron number (**f**) are also not altered in response to LRRK2 inhibition. No significant response was seen when compared with vehicle-treated neurons by one-way ANOVA with Dunnett’s multiple comparison test (**d**) or by Kruskal-Wallis test followed by Dunn’s multiple comparison test (**e**) and (**f**). (*N* = 9 biological replicates). Means + s.e.m.; all values are normalized to neurons treated with α-synuclein PFFs and DMSO. Scale bars = 50 μm

**Fig. 5:**
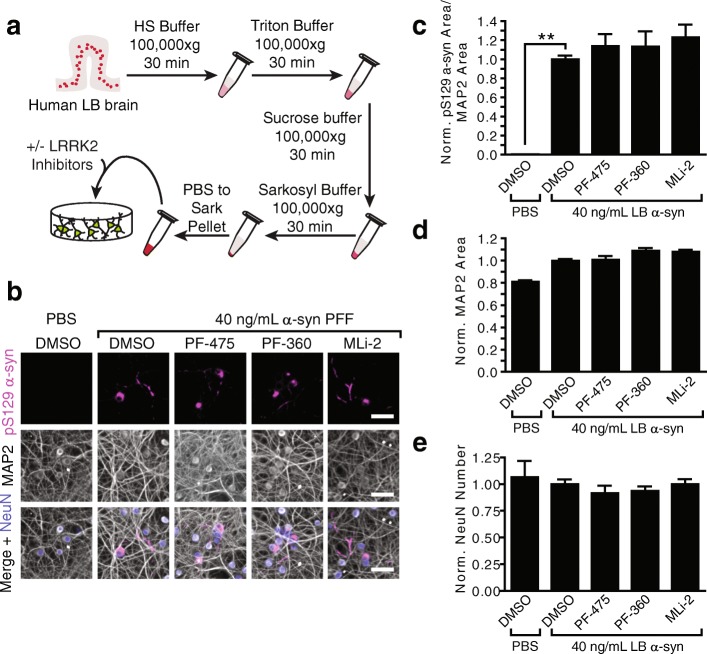
LRRK2 inhibition does not alter pathology induced by human Lewy body α-synuclein. **a** A schematic representation of pathological α-synuclein purification from human cortical tissue. **b** Primary hippocampal neurons from CD1 pups were treated with 100 nM LRRK2 inhibitors or a vehicle control. Two days later, neurons were treated with 40 ng/mL human LB α-synuclein and allowed to age a further 14 days prior to fixation and staining for pS129 α-synuclein (magenta), MAP2 (gray) and NeuN (blue). No large differences can be observed in the type or abundance of α-synuclein pathology. **c** Quantification of α-synuclein pathology reveals an increase in neurons treated with LB α-synuclein compared to those treated with PBS (**p < 0.01), but no change in response to LRRK2 inhibition. MAP2 area (**d**) and neuron number (**e**) are also not altered meaningfully in response to LRRK2 inhibition. (*N* = 11–12 biological replicates treated with LB α-synuclein from 4 separate cases (2 AD, 1 PDD, 1 DLB). Means + s.e.m.; all values are normalized to neurons treated with LB α-synuclein and DMSO. Scale bars = 50 μm

**Fig. 6:**
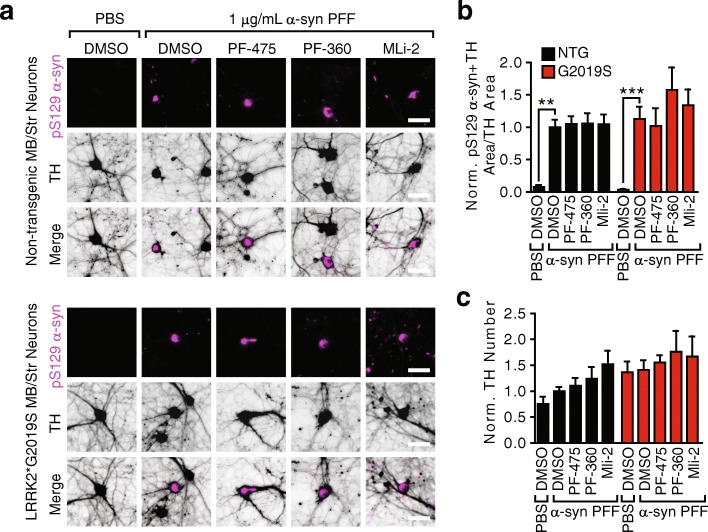
Neither G2019S LRRK2 expression nor LRRK2 inhibition alters α-synuclein pathology in midbrain neurons. **a** Primary midbrain/striatum cultures from NTG or G2019S pups were transduced with α-synuclein PFFs and allowed to age a further 14 days prior to fixation and staining for pS129 α-synuclein (magenta) and TH (gray). The neurons were additionally treated with 30 nM LRRK2 inhibitors PF-475, PF-360, or MLi-2 2 days prior to transduction and fed with media containing inhibitors each week thereafter. **b** Quantification of α-synuclein pathology in TH+ neurons shows no effect of G2019S LRRK2 expression or LRRK2 inhibitor treatments by 2-way ANOVA (**p < 0.01, ***p < 0.001 for PBS- compared to PFF-treated neurons by Dunnett’s multiple comparison test). **c** The number of TH+ neurons showed no significant response to treatment by Kruskal-Wallis test followed by Dunn’s multiple comparison test. (*N* = 6–9 biological replicates). Means + s.e.m.; all values are normalized to NTG neurons treated with α-synuclein LB material and DMSO. Scale bars = 50 μm

All statistical analyses were done in GraphPad Prism. The analysis used for each data set is described in the accompanying figure legend. More specifically, for experiments directly comparing only NTG and G2019S neurons (Fig. 1), unpaired t-tests with Welch’s corrections were performed. For experiments in which various inhibitors were compared, one-way ANOVAs with Dunnett’s multiple comparison test (Figs. 3 and 4b, d) or Kruskal-Wallis tests with Dunn’s multiple comparisons tests (Figs. 4b, e, f and 5) were performed. Experiments which included both NTG and G2019S neurons and various treatment conditions (Figs. 2 and 6), we performed two-way ANOVAs with Dunnett’s multiple comparison test to compare treatment type and Sidak’s multiple comparison test to evaluate the difference between genotypes.

## Results

### G2019S LRRK2 expression does not affect soluble or insoluble α-synuclein in hippocampal neurons

To establish a model in which the effect of LRRK2 mutations on α-synuclein could be observed, we used a transgenic BAC mouse which expresses mouse LRRK2 with the familial G2019S mutation under the endogenous LRRK2 promoter (B6.Cg-Tg(Lrrk2*G2019S)2Yue/J, [19]). This allows overexpression of the mutated LRRK2 in a similar pattern to endogenous LRRK2 [35]. Upon initiation of breeding, it was discovered that the mice have multiple inserts of the transgene and were heterozygous at all loci, resulting in non-transgenic pups upon inbreeding in a stochastic manner. To ensure homogeneity of cultures and reproducibility of results, the mice were bred to stable homozygosity as confirmed by quantitative PCR and outbreeding. The genotype of all animals used for culture was confirmed by quantitative PCR. We cultured primary hippocampal neurons from the G2019S LRRK2 (G2019S) or C57BL/6 J (NTG) control animals. G2019S neurons express ~ 25-fold elevated LRRK2, resulting in ~ 50-fold increase in phosphorylated LRRK2 at residue Ser935 (pS935), a readout for LRRK2 activity [6, 7, 26] (Fig. 1a, b). Despite dramatic elevation in LRRK2 expression and activity, soluble α-synuclein levels were unchanged (Fig. 1a, b). We induced α-synuclein pathology in these cultures as described previously [12] by adding 2.5 μg/mL recombinant mouse α-synuclein pre-formed fibrils (PFFs) to the cultures at 7 days in vitro (DIV) and allowing the neurons to develop pathology for a further 14 days post-transduction (DPT). The protein from neurons was then sequentially extracted in 1% TX-100 followed by 2% SDS, allowing the separation of pathological, insoluble α-synuclein from soluble α-synuclein. Both NTG and G2019S neurons accumulated insoluble α-synuclein phosphorylated at residue Ser129 (pS129) to a similar extent (Fig. 1a, c). Additionally, the autophagy receptor p62, which decorates pathological inclusions, including Lewy bodies in PD, accumulates to a similar extent in both genotypes (Fig. 1a, c). Thus, a dramatic elevation in LRRK2 activity does not alter pathological α-synuclein accumulation at 14 DPT.

### G2019S LRRK2 hippocampal neurons show mild, reversible elevation in α-synuclein pathology at 21 DPT

However, it has recently been demonstrated that G2019S neurons may show time-dependent changes in α-synuclein pathology that don’t become apparent until 18 DPT [32]. Therefore, we cultured NTG and G2019S neurons, treated at 7 DIV with 1 μg/mL α-synuclein PFFs, and allowed the neurons to develop pathology for 21 DPT. In addition, we treated the neurons 2 days prior to transduction with two validated LRRK2 inhibitors, PF-06447475 (PF-475) and PF-06685360 (PF-360) at 5, 30, and 120 nM. At 21 DPT, the neurons were fixed and stained for pS129 α-synuclein, MAP2 (a somatodendritic marker), and NeuN (a marker of neuronal nuclei). Both NTG and G2019S neurons develop robust neuritic and cell body pathology in comparison to neurons treated with PBS as a vehicle control which never develop pS129 α-synuclein inclusions (Fig. 2a, b). We were able to detect a mild elevation of α-synuclein pathology in the G2019S neurons, which was reversible with 30 and 120 nM PF-360 (Fig. 2a, b, c). In every other respect measured, NTG and G2019S neurons responded similarly to α-synuclein PFF treatment. The area covered by MAP2 and the number of neurons were not different between the two genotypes (Fig. 2d, e), and the mild elevation in pathology did not induce further toxicity. In addition, at no concentration did the LRRK2 inhibitors affect α-synuclein pathology, MAP2 area or neuron number in the NTG neurons indicating that there is no apparent relationship between LRRK2 activity in NTG neurons and α-synuclein pathology. We conclude that, while interesting, the observed elevation in α-synuclein pathology in the G2019S neurons is too mild to make this a valuable screening tool for LRRK2-dependent phenotypes.

### LRRK2 inhibitors do not alter α-synuclein pathology in wildtype neurons

The lack of an effect of LRRK2 inhibitors on α-synuclein pathology in NTG neurons was surprising given previous publications showing a reduction of α-synuclein pathology in response to both LRRK2 inhibition [32] and knockdown with anti-sense oligonucleotides [38]. While LRRK2 inhibitors are initially being tested in patients with LRRK2 mutations, there is speculation that if LRRK2 is a fundamental protein in PD pathogenesis, LRRK2 inhibitors may provide clinical benefit for those without LRRK2 mutations. Therefore, we sought to investigate LRRK2 in wildtype neurons from a second, distinct strain of mice (CD1). We first sought to validate the effect of LRRK2 inhibitors on LRRK2 activity in these neurons and further test these inhibitors on insoluble, pathological α-synuclein accumulation. CD1 hippocampal neurons were treated with LRRK2 inhibitors at 5 DIV and transduced with 2.5 μg/mL α-synuclein PFFs at 7 DIV. Neurons were harvested by sequential detergent fractionation at 14 DPT. We were able to confirm a robust ~ 75% inhibition of LRRK2 S935 phosphorylation with 30 nM PF-475 or PF-360 (Fig. 3a, b). PF-475 also modestly reduced total LRRK2 levels (Fig. 3a, b). However, inhibition of LRRK2 resulted in no reduction in insoluble α-synuclein, pS129 α-synuclein, or p62 (Fig. 3a, c). Instead, pS129 α-synuclein was slightly, but significantly elevated by LRRK2 inhibitor treatment (Fig. 3a, c).

We sought to further validate this finding with a larger family of LRRK2 inhibitors at increased concentrations that would leave no residual LRRK2 activity. We chose an additional, validated LRRK2 inhibitor (MLi-2) for further investigation and added these inhibitors at concentrations ranging from 30 to 300 nM to CD1 neurons. Use of these inhibitors resulted in near complete depletion of pS935 LRRK2 (Fig. 4a, b). The neurons were then transduced with α-synuclein PFFs and fixed at 14 DPT. No LRRK2 inhibitor, at any concentration, altered α-synuclein pathology (Fig. 4c, d), MAP2 area (Fig. 4c, e) or neuron number (Fig. 4c, f).

We then tested whether inhibition of LRRK2 activity can alter α-synuclein pathology induced by a means other than PFFs. We have recently demonstrated the ability of LB α-synuclein purified from human brain to induce pS129 α-synuclein pathology in WT neurons [24]. Cortical gray matter from brains with high LB burden were sequentially extracted with high salt, Triton X-100, sucrose, and sarkosyl buffers, yielding a final pellet enriched in LB α-synuclein. This pellet was then suspended in phosphate-buffered saline by sonication, yielding a final concentration of α-synuclein from 7.5–22.4 μg/mL, Fig. 5a, Table 1). As before, primary hippocampal neurons were treated with LRRK2 inhibitors at 5 DIV followed by treatment with 40 ng/mL LB α-synuclein two days later. This is the maximum concentration that neurons can be treated with due to the relatively low concentration of α-synuclein in these preps. Neurons were fixed and stained 14 days after the addition of LB α-synuclein. The induced pathology is sparser than that induced by PFFs due to the lower concentration of α-synuclein (Fig. 5b). The pathology induced by LB α-synuclein and neuron health were not meaningfully altered by LRRK2 inhibition (Fig. 5b, c, d, e), consistent with PFF treatment.

**Table 1:** Clinical Information of the cases used for the Extraction of Pathological alpha-synuclein

Case No.	Clinical Diagnosis	Pathological Diagnosis	Race	Sex	Age of Disease onset	Age at Death	PMI (h)	Brain Region Used	α-Synuclein (μg/ml)
1	AD Probable	AD/DLB	White	Female	52	62	11	Middle Frontal Gyrus	20.86
2	CBS	AD/DLB	White	Female	45	55	15	Middle Frontal Gyrus	22.43
3	DLB	DLB	White	Male	76	83	6	Cingulate Gyrus	7.55
4	PDD	PDD	White	Male	60	72	19	Cingulate Gyrus	8.28

*PMI* postmortem interval

### Dopaminergic neurons show no change in α-synuclein pathology in response to G2019S LRRK2 overexpression or LRRK2 inhibition

All the experiments to this point were performed in primary hippocampal neuron cultures. These cultures develop robust α-synuclein pathology and can be obtained in an abundance suitable for both immunocytochemistry and biochemistry. However, they are not the neurons most affected in PD patients. In order to address whether we were missing a phenotype that is dependent on expression of mutant LRRK2 in dopaminergic neurons, we developed ventral midbrain and striatum primary neuron co-cultures. When treated with α-synuclein PFFs at 7 DIV and allowed to age 14 DPT, dopaminergic neurons positive for TH develop cell body and neuritic pS129 α-synuclein pathology (Fig. 6a). We treated midbrain/striatum co-cultures from NTG and G2019S pups with 30 nM LRRK2 inhibitors or vehicle control at 5 DIV. The neurons were then treated with α-synuclein PFFs at 7 DIV and fixed 14 DPT and stained for pS129 α-synuclein and TH. Both cultures develop a similar amount of α-synuclein pathology in TH+ neurons, and none of the LRRK2 inhibitors changed the level of pathology in either NTG or G2019S neurons (Fig. 6a, b). Further, α-synuclein PFFs were not more toxic to G2019S dopaminergic neurons, and LRRK2 inhibitors did not significantly improve survival of TH+ neurons (Fig. 6c).

## Discussion

In this study, we utilized primary neuron cultures to assess the status of α-synuclein pathology in relation to LRRK2 activity. We performed biochemical and immunocytochemical analysis of primary hippocampal and midbrain neurons cultured from two strains of wildtype mice and a mouse expressing LRRK2 with the familial G2019S mutation. To further manipulate LRRK2 activity, we used three validated LRRK2 inhibitors. In none of these assays did we observe more than a mild LRRK2-dependent alteration in α-synuclein pathology.

The LRRK2*G019S neurons we describe have ~ 25-fold elevated LRRK2 levels and ~ 50-fold elevated kinase activity (Fig. 1a, b). Despite this dramatic overexpression of LRRK2, there is no apparent effect on the neuron health or viability (Figs. 2, 4, 5, and 6). This is in contrast with early research using viral overexpression of LRRK2 [8, 18], but in keeping with the many transgenic models of LRRK2 that have since been generated [3, 19, 20, 37]. While there is strong support that overexpression of LRRK2 does not dramatically alter cell health, the effect of LRRK2 on α-synuclein has been unclear.

We induced pathological α-synuclein inclusion formation in NTG and G2019S neurons through addition of recombinant α-synuclein PFFs or human brain-derived LB α-synuclein. We designed our initial experiments to test the relationship between LRRK activity and α-synuclein at 14 DPT, before the onset of neurodegeneration. We found that there was no alteration of α-synuclein pathology in G2019S neurons. A recent publication using a similar model showed a mild elevation in α-synuclein in G2019S hippocampal neurons at 18 DPT, while detecting no difference at 7 DPT [32]. We found that at 21 DPT, we did observe a mild enhancement of α-synuclein pathology in G2019S neurons that was responsive to LRRK2 inhibition (Fig. 2). At this timepoint, neurons have begun degenerating, and while we see no LRRK2-dependent difference in degeneration, it is unclear if it is the neurodegenerative process that results in a mild elevation of α-synuclein pathology in G2019S neurons.

To further explore the role of endogenous LRRK2 in α-synuclein pathogenesis, we cultured wildtype hippocampal and midbrain neurons, and showed using biochemistry and immunocytochemistry that LRRK2 kinase inhibition is unable to alter induced α-synuclein pathology (Figs. 3, 4, 5, and 6). These findings are in sharp contrast to recent reports that LRRK2 inhibitors [32] or LRRK2 protein reduction by anti-sense oligonucleotides [38] are able to ameliorate α-synuclein pathology in wildtype neurons. To ensure the validity of our results, we used both biochemical extraction of pathological α-synuclein as well as immunocytochemistry. Pathology induced by both recombinant α-synuclein PFFs and human LB α-synuclein were resistant to alteration by LRRK2 inhibition. We also used three validated LRRK2 inhibitors, representing different classes of compounds. All immunocytochemical quantification of α-synuclein was normalized to MAP2 to ensure no effect of cell density. Our results in wildtype hippocampal neurons generated from two strains of mice in addition to dopaminergic midbrain neurons give us increased confidence that the lack of LRRK2-dependent phenotypes we see are valid.

LRRK2 is the most common cause of inherited PD. Even so, only around 30% of those with G2019S mutation in LRRK2 will go on to develop PD [22]. It is therefore probable that LRRK2 mutations exacerbate an extant predisposition to PD. Another hypothesized determinant of susceptibility to PD is the misfolding of α-synuclein within aging brains. It is logical to assume that challenging neurons with both misfolded α-synuclein and mutated LRRK2 would be sufficient to drive an enhanced pathogenic process. While there is compelling evidence that this may be true in mouse models [3, 5, 32], we are unable to generate a robust model of LRRK2-dependent α-synuclein pathogenesis in primary neuron cultures. There are several possible reasons that in vivo models cannot be recapitulated in cell culture. Neuron cultures are inherently closed systems. Therefore, if LRRK2 activity can affect the spread of α-synuclein pathology through the brain, this effect would not be captured in a dish. Primary neuron cultures also lack all the cell types and complex synaptic organization present in vivo. Indeed, LRRK2 is highly expressed in astrocytes [13] and neuroinflammation has been implicated in LRRK2-dependent phenotypes [3]. It is also possible that LRRK2 is not critical in the early stages of pathology formation, and only during later stages of disease progression does LRRK2 activity become important. This is supported by our finding that α-synuclein pathology is only elevated in G2019S neurons 21 days after transduction, after the onset of neurodegeneration. In addition, patients with LRRK2 mutations do not have an earlier onset of disease than patients with sporadic onset [11], suggesting that LRRK2 does not speed the disease process, as triplication of α-synuclein does [27]. While α-synuclein inclusions are common in PD patients with LRRK2 mutations, not all LRRK2 patients have α-synuclein pathology and a substantial portion of LRRK2 patients also have tau inclusions [25]. Future studies of the pathological spread of α-synuclein and tau in vivo are warranted to better understand the underlying etiology of LRRK2 mutations in PD.

## Conclusions

There is substantial genetic and pathological data indicating that mutations in LRRK2 lead to an increased susceptibility to PD, and LRRK2 inhibitors are currently in clinical trials for the treatment of PD. Yet, how mutations in LRRK2 predispose patients to PD is still unclear. In this study, we show that in primary neurons, only after the onset of neurodegeneration does G2019S LRRK2 elevate α-synuclein pathology. Further, while LRRK2 inhibitors can rescue this elevated pathology, they show no benefit to wildtype neurons. Future studies of the link between LRRK2 mutations and the pathophysiology of PD will be critical to inform clinical studies of LRRK2 inhibitors.
